# The Heart in the Mind: A Systematic Review and Meta-Analysis of the Association Between Theory of Mind and Cardiac Vagal Tone

**DOI:** 10.3389/fphys.2021.611609

**Published:** 2021-07-09

**Authors:** Marta Zammuto, Cristina Ottaviani, Fiorenzo Laghi, Antonia Lonigro

**Affiliations:** ^1^Department of Social and Developmetal Psychology, Sapienza University of Rome, Rome, Italy; ^2^Department of Psychology, Sapienza University of Rome, Rome, Italy; ^3^Functional Neuroimaging Lab, IRCCS Fondazione Santa Lucia, Rome, Italy; ^4^Department of Human Sciences, European University of Rome, Rome, Italy

**Keywords:** heart rate variability, vagal tone, parasympathetic, theory of mind, social cognition, meta-analysis, systematic review

## Abstract

Theory of mind (ToM) is the human ability to infer the mental states of others in order to understand their behaviors and plan own actions. In the past decades, accumulating evidence has shown that heart rate variability (HRV), an index of parasympathetic control of the heart, is linked to behavioral regulation, social competence, and social cognition abilities, all implicated—to some extent—in ToM. This study aims to systematically review and meta-analyze the available studies, investigating the relation between ToM and HRV in typically developing people. Six studies were eligible for the meta-analysis, yielding a significant association between HRV and ToM of a small-to-medium effect size (*g* = 0.44). This result was not influenced by publication bias. Due to the small number of studies eligible for the meta-analysis, it was not possible to test for the effect of categorical moderators. The moderating role of sex and quality of the studies was examined by meta-regression analysis. Moderation analysis did not yield any significant effect; however, at a descriptive level, studies yielding the largest effect size were characterized by the use of high frequency-HRV assessment at rest and the Reading the Mind in the Eyes Test to evaluate ToM abilities. The results preliminarily suggest that tonic HRV might be used as an indicator of the ability to understand the content of mind of others.

## Introduction

### Theory of Mind and Social Behavior

Theory of mind (ToM) is a component of social cognition (Arioli et al., 2018) and it denotes the ability to infer the inner states of others, such as doubts, purposes, intentions, beliefs, and thoughts (Frith and Frith, 2003; Flavell, 2004). Since 1978, when Premack and Woodruff (1978) introduced the expression of ToM in their pioneering study with primates, the interest toward this ability has largely grown, allowing us to recognize its great impact on social life. During social exchanges, people must constantly impute mental states of their interlocutors in order to correctly attribute a meaning to their behaviors.

As a whole, good ToM abilities facilitate the engagement in social behavior. The majority of studies documented a positive relation between ToM and social relationships (for a review, see Hughes and Leekam, 2004), peer popularity (for a meta-analysis, see Slaughter et al., 2015), prosocial behavior (for a meta-analysis, see Imuta et al., 2016), and social adjustment (Capage and Watson, 2001; Banerjee and Watling, 2005). However, other investigations—especially those regarding bullying, machiavellianism, and conduct disorders (Astington, 2003; Lonigro et al., 2014)—pointed out a “dark side” of ToM, revealing that the relation between ToM and prosocial conduct is far from straightforward and simple. High levels of ToM abilities at times may lead to negative outcomes, as demonstrated by Hughes et al. (1998) in their study. The authors found that controls outperformed children with conduct disorders on an affective perspective taking task but no differences emerged between the groups on a deception task. Evidence on the maleficent use of ToM has led Astington to postulate that “although false-belief understanding is necessary for some social behaviors, it is never sufficient to guarantee the performance of such behavior” (Astington, 2003, p. 33). In other words, some people may decide to use ToM for prosocial purposes, namely, nice ToM, but others may use it for antisocial purposes, such as threatening, manipulating, or bullying (nasty ToM; Ronald et al., 2005; McEwen et al., 2007).

At present, it is not completely clear why children with the same ToM abilities may engage in different social behaviors. Ronald et al. (2005) argued that the social use of ToM abilities is difficult to understand because the ToM tasks that are traditionally used (e.g., false-belief understanding) are neutral with respect to the nature of the social behaviors involved. In fact, in daily life, few situations requiring ToM are neutral. In this regard, while some researchers have directed their attention to the role of moral disengagement (Ettekal and Ladd, 2020), others have focused on empathy, in particular, affective or emotional empathy pointing to its significant impact on social behavior (e.g., Lonigro et al., 2014). ToM and empathy are fundamental constructs for successful social interaction and are often considered interchangeable. ToM refers to the ability to infer the cognitive (e.g., thoughts and beliefs) and affective contents (e.g., desires and emotions) of unobservable mental states. Such ability requires the representation of a propositional attitude, namely, meta-representation. Cognitive empathy is commonly conceptualized as the ability to reason about the emotions of others, whereas emotional empathy concerns engaging in a congruent and immediate response with such an emotional state. Thus, affective empathy encompasses empathetic concern, sympathy, or compassion for the emotions of others (Schurz et al., 2021). Dvash and Shamay-Tsoory (2014) have proposed a model in which ToM and cognitive empathy overlap, involving the same neural networks, while affective empathy represents a different system (Tholen et al., 2020). Notably, the development of mentalizing abilities follows a distinct trajectory from that of affective empathy (Sebastian et al., 2012; Slaughter et al., 2015). In this study, we adopted the same approach used by previous meta-analytic studies on ToM among typically developing people (Slaughter et al., 2015; Imuta et al., 2016), thus excluding affective empathy.

### The Polyvagal Theory and the Social Engagement System

From the early 2000s, the field of behavioral medicine has identified several physiological correlates of different aspects of social cognition. For example, within the framework of the polyvagal theory (Porges, 2003), parasympathetic influences on emotional and socio-cognitive processes have been deeply investigated with the ultimate aim to explain social behavioral development (Porges et al., 1996; Porges, 2001). This theory considers cardiac vagal tone as a behavior regulator responding to external and internal environmental cues. In detail, Porges theorized that two vagal circuits regulate affiliation and social behavior, i.e., a more archaic branch called *dorsal vagal*, which regulates visceral and homeostatic functions, and a more recent branch composed of the myelinated vagal fibers (*ventrovagal*) that form the neural substrate of the social engagement system (Porges, 2007).

An indirect, well-validated measure of vagal modulation of the heart is heart rate variability (HRV; Kuo et al., 2005), a measure of beat-to-beat variations in heart rate over time. Empirical evidence has shown that higher resting HRV is linked to behavioral regulation (e.g., Calkins, 1997), social behavior (e.g., Stifter and Corey, 2001), emotional regulation adaptive strategies (e.g., Cole et al., 1996; Santucci et al., 2008), and prosocial traits (e.g., Miller et al., 2017) in developmental years. As a result, HRV is likewise linked to prosociality (e.g., Kogan et al., 2014), cooperative behavior (e.g., Beffara et al., 2016a), compassion (Di Bello et al., 2020 for a meta-analysis), emotional recognition (e.g., Quintana et al., 2012), memory (e.g., Mattarozzi et al., 2019), empathy (e.g., Lischke et al., 2018), and social connectedness (e.g., Kok and Fredrickson, 2010) in adulthood. Only a few studies, however, investigated phasic HRV responses during tasks assessing ToM-related abilities, although this would represent an important measure of momentary physiological concomitants of such abilities (Park et al., 2014).

### Theory of Mind and HRV: The Present Study

In the past decade, researchers investigated whether inter-individual differences in vagally mediated HRV would be associated with inter-individual differences in mindreading ability. As a matter of fact, autonomic dysfunctions have been found in atypical development syndromes, namely, autism spectrum disorder (ASD) (Benevides and Lane, 2015; Saghir et al., 2017) and schizophrenia (Jáuregui et al., 2011), which are characterized by social cognition difficulties. In one of the few experimental studies, Colzato et al. (2017) observed that transcutaneous vagus nerve stimulation improves ToM, measured with the Reading the Mind in the Eyes Test, during which participants must infer what a person is thinking or feeling only by looking at photographs of the eye region of the face, with the possibility to choose the correct answer among four words (Baron-Cohen et al., 2001a).

An association between better social cognition abilities and higher HRV, however, has not always been found (e.g., see Hamilton et al., 2014). This may be due to the fact that “social cognition” is a comprehensive term that includes different abilities, including ToM (Baars and Gage, 2013), which are probably differently associated with vagal modulation of the heart.

Adopting the same approach used by previous meta-analyses on ToM (Slaughter et al., 2015; Imuta et al., 2016), this study focused on tasks that evaluate the ability to understand the mental states of others (e.g., intentions, desires, thoughts, and knowledge). Thus, this study aimed to systematically review and meta-analyze the available studies examining the relation between (tonic and phasic) HRV and ToM in typically developing people. The moderating role of sex was examined, due to sex differences in autonomic function, with females showing greater high-frequency HRV (HF-HRV) (for a meta-analysis, see Koenig and Thayer, 2016). In contrast, sex differences in ToM appear controversial. While in some studies males and females had the same performance on ToM tasks (Di Tella et al., 2020), in other studies females had better performance compared with males during preschool and elementary school years (Charman et al., 2002; Walker, 2005; Calero et al., 2013), adolescence (Gabriel et al., 2021), and adulthood (Wacker et al., 2017).

## Methods

### Search Strategy

A systematic analysis of the international literature was carried out by searching articles on PubMed, PsycINFO, and Web of Science databases. The last search was conducted on January 22, 2020. The results were restricted to “from the 1980s,” considering the year in which ToM conceptualization appeared in the literature for the first time (Wimmer and Perner, 1983). The keywords used in the literature search were as follows: “heart rate,” “autonomic nervous system,” “heart rate variability,” “root mean square successive difference,” “respiratory sinus arrhythmia,” “vagal,” “vagus,” “sympathetic,” “psychophys^∗^,” “parasympathetic” (see Ottaviani et al., 2016) in combination with each of the following key words, using the Boolean connector and, “theory of mind,” “ToM,” “mentalizing,” “mindreading,” “mind understanding,” “social understanding,” “mental representations,” “mental states,” “false belief,” “perspective taking,” and “social cognition” (see Imuta et al., 2016).

### Eligibility Criteria

In agreement with the above-mentioned definition provided for ToM, we included studies in which ToM was evaluated with first-order and/or second-order false belief tasks, perspective taking tasks, and other tasks related to the ability to infer the mental states of others. The studies had to include HRV assessment in typically developing participants. The reports written in English and Italian were considered. Single case and review studies were excluded.

### Selection and Coding of Primary Studies

**FIGURE 1:**
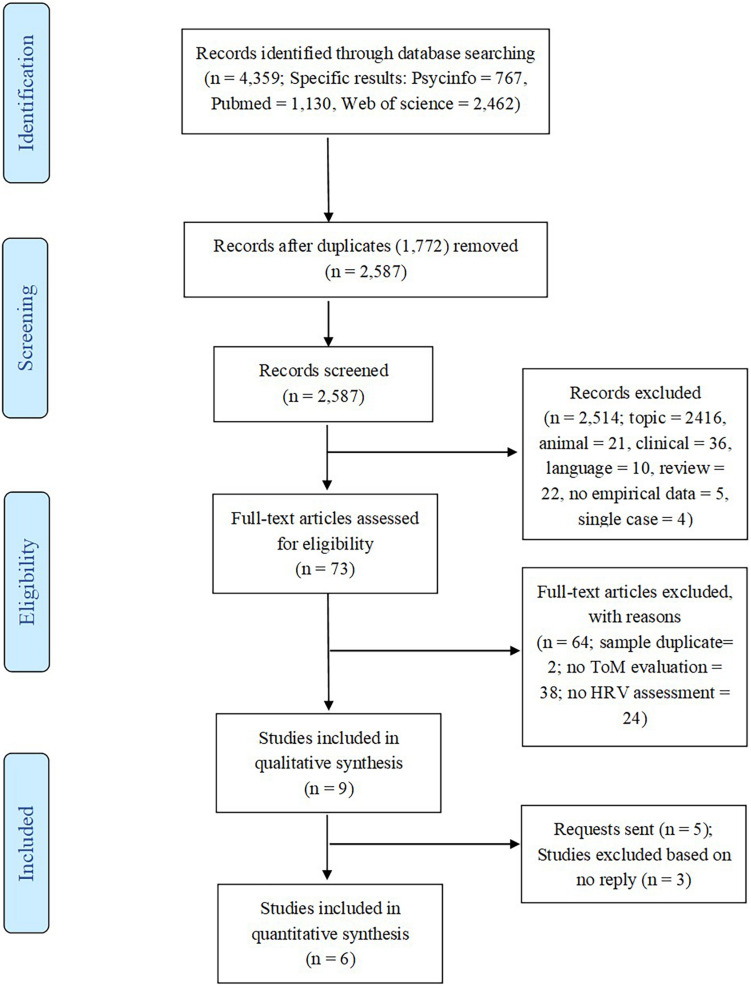
PRISMA diagram (Moher et al., 2009).

The review was conducted according to the PRISMA statement methodology (Moher et al., 2009; Crocetti, 2016). Once the 1,772 duplicates were removed, the remaining 2,587 records were independently screened by the first (MZ) and the last (AL) authors. Inter-coder agreement was very high at the abstract stage (% agreement = 99.23%; Cohen’s kappa = 0.84). When the independent coders examined the full text of the 74 articles identified as eligible, the agreement between the coders remained high (% agreement = 97.26%; Cohen’s kappa = 0.77). Disagreements were resolved through discussion. Figure 1 illustrates the reasons for excluding 65 studies: 9 studies were included in the qualitative synthesis and 6 of them were included in the quantitative analysis.

The data from the studies of interest were extracted and inserted in a structured dataset sheet. According to the PICOS procedure (Liberati et al., 2009), names of authors, year of publication, number of participants,% of females, mean age, the country where the study was conducted, precautions taken before the HRV assessment, HRV parameters, assessment duration, ToM task(s), quality of the study, and main results were extracted. All the authors reviewed the summary table of the included studies. Only papers (published or sent by the authors) that provided the data needed to compute the correlation between measures of ToM and HRV were included in the meta-analysis. Zero-order correlations were preferred to compute effect sizes. The quality of selected studies was assessed with a modified version of the Newcastle–Ottawa Scale (Wells et al., 2011) previously adopted in a meta-analysis on psychophysiological measures (e.g., Ottaviani et al., 2016). The scores are comprised between 0 and 9, and a higher score is indicative of a higher quality of the study.

### Strategy of Analysis

Effect sizes were computed with the meta-analytic ProMeta 3 software (IDo Statistics-Internovi, Italy) using the random-effects model. Hedges’ *g* was preferred over Cohen’s *d* due to the small number of studies included in the meta-analysis. Heterogeneity across studies was estimated with the *Q* statistic and the *I*^2^ index. Sex and the quality of studies were tested as possible moderators using the meta-regression analysis. Publication bias was estimated by using the Egger’s linear regression method and Kendall’s tau.

## Results

The flowchart in Figure 1 reports the study selection process and illustrates the results in each step. Unpublished studies were not included in the meta-analysis because (i) dissertations did not include correlational data; (ii) the results found in conference papers were the same as reported later in published articles (coded as “sample duplicate” in Figure 1); and (iii) the authors did not respond to data requests.

### Study Selection and Characteristics of Included Findings

Nine articles were included in the qualitative systematic review (see Table 1). All study samples were composed of adults, except for one that involved children (Kushki et al., 2014).

**TABLE 1:** Summary of the studies included in the systematic review and in the meta-analysis.

Study	Sample	*N*	Age M (SD)	Females	Country	HRV measure	Precautions	Assessment	ToM task	Relation between HRV and ToM performance
1. Cugnata et al. (2018)	TD	91	26.78 (18–52)	48.35%	Italy	RMMSD	n.s.	RS	RMET	Effect of item difficulty and physiological activation (*p* = n.s.)
2. Deuter et al. (2018)*	TD	90	23.5 (3.5)	50%	Germany	RMMSD	No medications	RS	MET	Cognitive empathy and HRV (*r* = −0.05, *p* = 0.65; sent by the authors)
3. Iorfino et al. (2016)*	CG	25	23.96 (2.19)	0%	Australia	HF-HRV	No alcohol and illicit substance 24 h prior to testing, and no smoking, food, and drinking 3 h before testing	RS and Task	RMET split version	HF at baseline and RMET score during no facial cooling (*p* = n.s.)
4. Jáuregui et al. (2011)*	CG	19	27.6 (6)	42.11%	Argentina	HF-HRV	Abstain from smoking for 2 h, no caffeinated beverages for at least 6 h, and no strenuous physical exercise for 24 h	RS and Task	RMET and Faux Pas Test (Stone et al., 1998)	Performance on the Faux Pas Test and HF-HRV during the test (*r* = 0.436, *p* < 0.05)
5. Kushki et al. (2014)	CG	34	12.5 (2.9)	44.1%	Canada	RSA	No medications and no premature birth	RS and Task	RMET-C	In response to the RMET-C, marginally larger decrease in RSA in the ASD group and larger RSA increase during recovery from the RMET-C (*p* = n.s.)
6. Lischke et al. (2017)*	TD	37	23.03 (3.56)	50%	Germany	HF-HRV	No medications and oral contraceptives	RS	RMET	Partial correlations# between HF-HRV and correctly identified positive (*r* = 0.379, *p* = 0.009)* and negative (*r* = −0.084; *p* = 0.305) states
7. Okruszek et al. (2017)	TD	25	35.9 (12.5)	48%	United Kingdom	RMMSD	No psychotropic medications	During daily activities	Hinting Task (Corcoran et al., 1995)	Performance and HRV parameters during daily life (8-h max recording period) (*p* = n.s.)
8. Quintana et al. (2012)*	TD	65	20.91 (6.16)	53.8%	Australia	HF-HRV	No caffeine, nicotine, and alcohol on the testing day. Not current use of antidepressants	RS (after Task)	RMET	HF-HRV at rest (adjusted for covariates) and RMET scores weighted for difficulty (*r* = 0.290, *p* < 0.05)
9. Varas-Díaz et al. (2017)*	CG	18	28.83 (3.36)	50%	Chile	RMMSD, HF-HRV	Refrain from coffee and medication 24 h before	RS and Task	RMET	% of RMET correct answers and (a) resting RMSSD (*r* = 0.381, *p* = 0.119)* and HF (*r* = 0.221, *p* = 0.379) and (b) RMSSD (*r* = 0.477, *p* = 0.045) and HF (*r* = 0.470, *p* = 0.049) during the task (sent by the authors)

*Studies/analysis included in the meta-analysis. TD, typical development; CG, control group; M, mean; SD, standard deviation; n.s., not specified; RSA, respiratory sinus arrhythmia; RMSSD, root mean square of successive differences; HF-HRV, high frequency-heart rate variability; RMET, Reading the Mind in the Eyes Test (Baron-Cohen et al., 2001a); RMET-C, The Reading the Mind in the Eyes Test-Child version (Baron-Cohen et al., 2001b); MET, Multifaceted Empathy Test (Dziobek et al., 2008); Faux Pas Test (Stone et al., 1998); Hinting Task (Corcoran et al., 1995); RS, Resting State; r, Pearson correlation coefficient; p, significance value. #Controlling for sex, age, and psychopathology.

Two studies (Cugnata et al., 2018; Deuter et al., 2018) were not included in the meta-analysis because of multilevel analyses. At present, the way to correctly extract the effect sizes from these types of analyses continues to be controversial (Selya et al., 2012). In the case of clinical studies (Jáuregui et al., 2011; Kushki et al., 2014; Iorfino et al., 2016; Varas-Díaz et al., 2017), we focused only on data related to the control group.

### Theory of Mind Evaluation

In studies linking ToM with HRV, the Reading the Mind in the Eyes Test (RMET; Baron-Cohen et al.,1997a,b; Baron-Cohen et al.,2001a,b) is mostly used. This visual test, which is traditionally used to measure ToM abilities, consists of 36 pictures of the eye region showing different emotions and mental states. Participants must infer the correct emotional/mental state in the image, choosing one of four words presented in the picture. The RMET scores are generally computed by summing the correct answers. Specifically, seven of the included studies (Jáuregui et al., 2011; Quintana et al., 2012; Kushki et al., 2014; Iorfino et al., 2016; Lischke et al., 2017; Varas-Díaz et al., 2017; Cugnata et al., 2018) used the RMET to evaluate ToM abilities as a component of social cognition. Among these, two studies conceptualized the RMET as an emotional recognition task, not referring to ToM abilities (Varas-Díaz et al., 2017; Cugnata et al., 2018). One of these studies (Kushki et al., 2014) examined a developmental sample and therefore used the child version of the test^1^ (Baron-Cohen et al., 2001b). All the authors calculated the scores considering the total of the correct answers except for one (Lischke et al., 2017) that computed the difference between the percentage of correctly identified positive relative to neutral states and the percentage of correctly identified negative relative to neutral states. Jáuregui et al. (2011) also used other tests besides the RMET, i.e., the Faux Pas Test^2^ (Stone et al., 1998; Baron-Cohen et al., 1999), the Baron-Cohen Faces Test^3^ (Baron-Cohen et al., 1997b), and the Happè ToM Story Test^4^ (Happé et al., 1999). The authors, however, reported only results on the relation among HRV, the Faux Pas Test, and the RMET (Jáuregui et al., 2011).

Deuter et al. (2018) administered a PC-assisted short-version of the Multifaceted Empathy Test (MET; Dziobek et al., 2008) to assess cognitive empathy intended as a social cognition measure. The participants were required to infer the mental state of the person in the photo and were asked to indicate the correct emotion from a list of four. The test consists of 30 picture stimuli with people in emotionally charged situations, and in this case (Deuter et al., 2018), the images were presented in 3 blocks of 10 picture stimuli to detect the cognitive components of empathy. The accuracy in the detection of emotional states is usually considered an index of “cognitive empathy” (Blair, 2008), conceptualized as the ability to create a theory about the mental state of others and take their perspective (Perry and Shamay-Tsoory, 2013).

Okruszek et al. (2017) used the Hinting Task (Corcoran et al., 1995) consisting of 10 short vignettes that are read out to the participants and left in front of the participants. The vignettes end with one of the characters giving a hint to the other character. The participant is asked what the character really meant with his/her assertion. An appropriate inference scores two points. If no inference is drawn, a second more obvious hint is added, and the participant is asked to infer the intention again. A correct response at this stage is given a score of 1, an incorrect response is given a score of 0, and the next item is introduced.

Notably, tests that involve faces stimuli (i.e., the Faces Test and the RMET) brought about phasic decreases in total HRV, while the Happè ToM Stories Test produced modest and significant increases in the SD of normal-to-normal intervals (Jáuregui et al., 2011). When the study carried out in children was considered, an increase in respiratory sinus arrhythmia (RSA) during the task and a decrease during recovery after the task were observed (Kushki et al., 2014).

Studies that did not use traditional ToM tasks (Okruszek et al., 2017; Deuter et al., 2018) failed to report an association between HRV and performance on (1) the MET (Dziobek et al., 2008), which requires inferring mental states from pictures with people in emotionally charged situations (Deuter et al., 2018), not presenting neutral background as in the RMET and (2) the Hinting Task (Corcoran et al., 1995), which is both a visual and a verbal ToM task, and it also involves other comprehension abilities (Okruszek et al., 2017).

To summarize, four out of the seven studies that used the RMET found positive and significant associations between HRV and ToM performance (Quintana et al., 2012; Lischke et al., 2017; Varas-Díaz et al., 2017; Cugnata et al., 2018), although different HRV indices were considered.

### Characteristics of HRV Assessment: Measures, Timing, and Precautions

Except for Kushki et al. (2014), who used RSA, the most commonly used index of HRV was HF-HRV (Jáuregui et al., 2011; Quintana et al., 2012; Iorfino et al., 2016; Lischke et al., 2017; Varas-Díaz et al., 2017). Okruszek et al. (2017); Cugnata et al. (2018), and Deuter et al. (2018) analyzed a time-domain component, namely, the root mean square of successive differences (RMSSD). Both indices are measures of vagally mediated HRV (Task Force of the European Society of Cardiology and the North American Society of Pacing and Electrophysiology, 1996).

In most studies, the authors recorded physiological activity for 5 min with a minimum of 4 and a maximum of 10 min in laboratory studies. Only one ambulatory study (Okruszek et al., 2017) assessed HRV during 8 h for 6 days of activities, including socializing moments.

Regarding the time of the day in which HRV was assessed, Jáuregui et al. (2011) and Quintana et al. (2012) recorded parasympathetic activity in the morning, while Varas-Díaz et al. (2017) assessed it from 3 to 6 p.m. and Iorfino et al. (2016) from 12 a.m. to 7 p.m.

Neurological, psychiatric, or psychological disorders, acute or persistent neurological or cardiovascular disease, and disability were excluded in all of the examined studies. Additional exclusion criteria were to refrain from medication in general (Kushki et al., 2014; Varas-Díaz et al., 2017; Deuter et al., 2018), oral contraceptives (Lischke et al., 2017), antidepressants (Quintana et al., 2012), psychotropic medication (Okruszek et al., 2017), antiparkinsonian anticholinergic agents, beta-blockers, and angiotensin-converting enzyme inhibitors (Jáuregui et al., 2011). Most of the authors asked participants to refrain from caffeinated beverages, food, and smoking before the testing session, from a minimum of 2 h (Jáuregui et al., 2011) to a maximum of 24 h (Varas-Díaz et al., 2017), and not to engage in physical activities (Jáuregui et al., 2011). Some authors also had alcohol consumption (Quintana et al., 2012; Iorfino et al., 2016) and illicit substances (Iorfino et al., 2016) as exclusionary criteria (see Table 1 for further methodological details).

Resting (i.e., tonic) HRV was mostly assessed, sometimes failing to show a significant association with performance on ToM tasks (Iorfino et al., 2016; Okruszek et al., 2017; Deuter et al., 2018). It has to be noted, however, that when time trajectories of phasic HRV in response to the task was examined, positive association between the performance on the RMET and RMSSD (Cugnata et al., 2018) and between performance on a verbal ToM test (Faux Pas Test) and HF-HRV (Jáuregui et al., 2011) were reported, respectively.

### Meta-Analysis on the Association Between HRV and ToM

Six of the nine studies included in the qualitative review were included in the meta-analysis as (i) they included correlational data on HRV and ToM (Jáuregui et al., 2011; Quintana et al., 2012; Iorfino et al., 2016; Lischke et al., 2017) or (ii) such data were provided by the authors on request (Varas-Díaz et al., 2017; Deuter et al., 2018)^5^.

**FIGURE 2:**
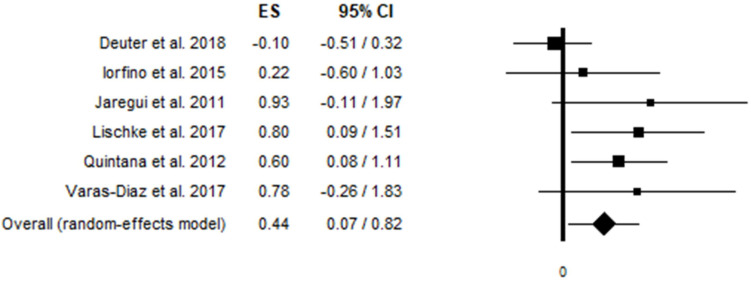
Forest plot for meta-analysis on the association between HRV values and ToM performance.

Analysis of the six studies (i.e., 255 participants; 119 females) showed a significant association between HRV and ToM performance [*g* = 0.44, 95% CI (0.07, 0.82), and *p* = 0.021], revealing a small-to-medium effect size^6^. Figure 2 shows the forest plot. No significant heterogeneity was detected *Q* (5) = 8.90, *p* = 0.113; *I*^2^ = 43.82. Egger’s test (intercept = 2.51, *t* = 1.92, and *p* = 0.127) and Kendall’s tau (*Z* = 0.56 and *p* = 0.573) did not detect the presence of publication bias.

Meta-regression analysis was computed considering sex as a continuous variable (i.e., % of women). The meta-regression analysis did not show a statistically significant role of sex in moderating the association between resting HRV and performance on ToM tasks (*p* = 0.712). The quality of studies did not emerge as a significant moderator (*p* = 0.988).

## Discussion

### Summary of Main Findings

The aim of the present systematic review and meta-analysis was to ascertain the relation between performance on tasks aimed at assessing the ability to infer mental states of others, namely ToM, and resting HRV. The effect size computed on the six studies with available data (Jáuregui et al., 2011; Quintana et al., 2012; Iorfino et al., 2016; Lischke et al., 2017; Varas-Díaz et al., 2017; Deuter et al., 2018) revealed a significant association of a small-to-medium size between tonic HRV and performance on ToM tasks. Neither sex nor the quality of the study moderated the relation between these two variables, likely due to the paucity of studies.

When considered individually, only two of the included studies were characterized by statistically significant results (Quintana et al., 2012; Lischke et al., 2017). In both studies, ToM was evaluated with the RMET, and HF-HRV was assessed as a measure of vagally mediated HRV. Lischke et al. (2017) evidenced a positive correlation between the identification of positive (but not negative) mental states of others and HF-HRV at rest. These findings are in line with the notion that individuals with high resting HF-HRV are more successful in initiating and maintaining social relationships than the individuals with low resting HF-HRV (e.g., Shahrestani et al., 2015).

A significant association between resting HRV and ToM performance also emerged when the RMET items were weighted for difficulty and HF-HRV values were adjusted for covariates, such as sex, body mass index, smoking habits, physical activity, and levels of depression, anxiety, and stress (Quintana et al., 2012). It has been reported that the RMET captures ToM deficits shown by people with ASD more than other tests do (Peterson and Slaughter, 2009) because it requires not only the identification of primary emotions from facial expression but also the recognition of mental states from a specific portion of the face. In fact, the understanding of inner states is more complex than the recognition of primary emotions, which has been more deeply investigated in relation to the vagal modulation of the heart (Bal et al., 2010; Park et al., 2012; Quintana et al., 2012; Beffara et al., 2016b; Lischke et al., 2017).

Two other studies that used the RMET failed to find a significant association with HRV (Iorfino et al., 2016; Varas-Díaz et al., 2017). In comparison with the previously examined studies, Iorfino et al. (2016) used the split version of the RMET, and Varas-Díaz et al. (2017) assessed vagally mediated HRV with time-domain measures (i.e., RMSSD). Other studies that obtained non-significant findings used the Faux Pas Test (Jáuregui et al., 2011) or the MET (Deuter et al., 2018).

In summary, the use of HF-HRV at rest and the RMET appear to best capture the relation between the vagally mediated HRV and ToM. Notably, when HRV and ToM were examined in children with ASD and controls (Baron-Cohen et al., 2001b), an atypical parasympathetic modulation in the ASD group during the RMET, but not at rest, was emerged (Kushki et al., 2014).

### Limitations and Future Directions

The main limitation of this study is the paucity of studies included in the meta-analysis; thus, caution is required when interpreting the results. For this reason, the impact of categorical moderators could not be considered.

Second, the current meta-analysis was not preregistered, and we are aware that protocol registration is a highly desirable practice that allows avoiding research duplications or overlaps, ensuring a careful study plan and research implementation promotion (Shamseer et al., 2015). However, Xu et al. (2019) compared meta-analyses with and without protocol, demonstrating that protocol registration was associated with better quality of reporting but not with improved methodological quality.

Third, the polyvagal theory, which together with the neurovisceral integration model (Thayer and Lane, 2000, 2009; Thayer et al., 2009) provided the theoretical background for this study, has been criticized for the weak empirical evidence on its phylogenetic basis. More specifically, Berntson et al. (2007) have argued that the smart vagus is already present not only in mammals but also across vertebrate species (e.g., cartilaginous fish). Moreover, these authors have pointed out the difficulty to clearly distinguish the dorsal motor nucleus and nucleus ambiguous contributions to human behavior, suggesting that the association between specific behavioral patterns (e.g., adaptive behavior, mobilization, and immobilization response) and the myelinated or unmyelinated vagus nerve appears to be misleading (Berntson et al., 2007).

Meanwhile, the results from several meta-analytic works suggested that vagal control is positively associated with social functioning (Shahrestani et al., 2014, 2015) and positive effect (Di Bello et al., 2020), as well as negatively correlated with psychopathological symptoms and conditions (e.g., Chalmers et al., 2014; Koenig et al., 2016; Ottaviani et al., 2016; Koch et al., 2019). More recently, Marmerstein et al. (2021) have questioned the link between vagal modulation and HRV, showing a lack of association between HRV measures (e.g., RMSSD, HF-HRV, and LF-HRV) and tonic vagal activity assessed in the left cervical vagus and with a respiratory vagal difference in rats with and without anesthesia. Conversely, other researchers have asserted that HF-HRV and other time-domain metrics (e.g., RMSSD) constitute a good measure of vagal activity in humans, as suggested by a seminal preclinical study on this topic (Ter Horst and Postema, 1997). Such opposite views represent a challenge for future research.

The neurovisceral integration model (Thayer and Lane, 2000, 2009; Thayer et al., 2009) provides an important description of the network that includes neural, visceral, and cardiac components, in regulating affective and social processes. Considering the recent formulation of the model (Smith et al., 2017), it is important to evaluate vagal modulation both at rest and when social cognition reasoning is engaged. In fact, based on the different levels of communication between the cortex system, neural afferents, and cardiac circuits, which are hierarchically organized, complex cognitive and emotive functions can be carried on only if basic processes are satisfied. Thus, we can view such integration capacity only during specific tasks that require the engagement of more complex networks. Resting HRV is generally mostly used as an index of physical and psychological wellbeing (Task Force of the European Society of Cardiology and the North American Society of Pacing and Electrophysiology, 1996). On the other hand, phasic fluctuations in HRV may reflect effortful emotional regulation during ToM tasks.

Finally, it seems that frontal lobe contributions to ToM might be important for representing mental states, whereas the parietal lobe role might be more specifically involved in reasoning about beliefs (Sabbagh, 2013). In future research, it would be useful to adopt measures of implicit ToM, such as the “Triangles Playing Tricks” (Heider and Simmel, 1944; Abell et al., 2000), which requires the understanding of the mental state of others using social intuition.

## Conclusion

The prevalence of studies included adult samples, used visual ToM tasks, and considered RMSSD and HF-HRV as measures of vagally mediated HRV. Given the variety of tasks used, replication is warranted using more consistent experimental methods. The assessment of vagally mediated HRV both at rest and during the ToM tasks is also warranted as it provides different and complementary information. Finally, longitudinal and experimental studies on the association between the ability to infer the mental states of others and vagally mediated HRV are needed, since conclusions on directionality cannot be drawn based on the existing data.

## Data Availability Statement

The raw data supporting the conclusions of this article will be made available by the authors, without undue reservation.

## Author Contributions

MZ, CO, FL, and AL conceived the idea. MZ and AL performed the systematic research. AL encouraged and supervised MZ’s work. All authors discussed the results and contributed to the final manuscript.

## Conflict of Interest

The authors declare that the research was conducted in the absence of any commercial or financial relationships that could be construed as a potential conflict of interest.

## Footnotes

1 Children were presented with a set of 28 photographs (selected from the adult version of the task) depicting the eye region of the face and were asked to choose the word that best described what the person was thinking or feeling from a total of 4 items (only 1 correct). Performance was measured as the number of correct responses (Baron-Cohen et al., 2001b).

2 The test contained 20 stories, half of which including a social faux pas. The stories were presented one at a time. The text of each story was printed on a single page and posed in front of the participant. The researcher read the story aloud and then asked a series of questions about it. The authors (Jáuregui et al., 2011) quantified correct detections of unintentional Faux Pas (i.e., hits), correct rejections (i.e., detection of non-Faux Pas stories), and control questions designed to assess the comprehension of the story (Stone et al., 1998; Baron-Cohen et al., 1999).

3 This task consisted of 20 photographs of the frontal face of an actress displaying different facial expressions, photographed under controlled and standardized conditions: 10 photographs displaying emotions such as happiness (repeated 2 times), sadness, anger (repeated 2 times), sorrow, surprise (repeated 2 times), disgust, and distress, and 10 photographs showing complex mental states, such as scheming, guilt, thoughtfulness, admiration, quizzically, flirtatiousness, boredom, interest (repeated 2 times), and arrogance. Each picture had two labels, giving participants a choice between two emotions (one correct and the other incorrect). The authors (Jáuregui et al., 2011) selected the label pairs out of a group of 20 emotions and arranged them pseudo-randomly. The participants were asked to identify the label that most appropriately described the “thinking or feeling” mental state of an actress (Baron-Cohen et al., 1997b).

4 The verbal test consisted of 16 short stories, half of which invoking ToM. The ToM stories involved the interaction between people and contained double bluff, mistakes, persuasion, or white lies, with two examples of each type presented. Each ToM story was followed by a question testing the ability of participant to make inferences about mental states of others, usually the intentions of actors (Happé et al., 1999).

5 Varas-Díaz et al. (2017) provided HRV and ToM values, and we calculated the Pearson’s correlation between RMSSD and HF at baseline and during the task and ToM score (% of correct answers). The results are reported in Table 1. In the meta-analysis, resting RMSSD was used, because all of the included studies used HRV values at rest with the exception of the study by Jáuregui et al. (2011).

6 The effect size was considered small, medium or large if equal to 0.20, 0.50, and 0.80, respectively (Cohen, 1988).
